# An efficient genomic prediction method without the direct inverse of the genomic relationship matrix

**DOI:** 10.3389/fpls.2022.1089937

**Published:** 2022-12-21

**Authors:** Hailan Liu, Chao Xia, Hai Lan

**Affiliations:** Maize Research Institute, Sichuan Agricultural University, Chengdu, Sichuan, China

**Keywords:** genomic prediction, GBLUP, genomic relationship matrix, randomized Haseman–Elston regression, preconditioned conjugate gradient

## Abstract

GBLUP, the most widely used genomic prediction (GP) method, consumes large and increasing amounts of computational resources as the training population size increases due to the inverse of the genomic relationship matrix (GRM). Therefore, in this study, we developed a new genomic prediction method (RHEPCG) that avoids the direct inverse of the GRM by combining randomized Haseman–Elston (HE) regression (RHE-reg) and a preconditioned conjugate gradient (PCG). The simulation results demonstrate that RHEPCG, in most cases, not only achieves similar predictive accuracy with GBLUP but also significantly reduces computational time. As for the real data, RHEPCG shows similar or better predictive accuracy for seven traits of the *Arabidopsis thaliana* F2 population and four traits of the *Sorghum bicolor* RIL population compared with GBLUP. This indicates that RHEPCG is a practical alternative to GBLUP and has better computational efficiency.

## Introduction

Currently, genomic prediction (GP) has been widely applied to many species, such as dairy cattle, dairy sheep, maize, and wheat (Pszczola et al., 2011; Duchemin et al., 2012; Crossa et al., 2014). For example, significant achievements have been made in the genetic improvement of dairy cattle *via* GP in many countries, such as the United States, Australia, Canada, New Zealand, and France (Hayes et al., 2009; Winkelman et al., 2015; García-Ruiz et al., 2016; Weller et al., 2017). Moreover, GP helps to optimize the breeding procedure when used with many other breeding technologies. For example, it can accelerate the selection of superior pure lines from the large numbers of those generated by doubled haploid (DH) technology, which is otherwise a significant problem in terms of the consumption of time and money (Wang et al., 2020). Additionally, GP can rapidly increase the frequencies of favorable alleles when combined with genome editing (GE) (Jenko et al., 2015; Bastiaansen et al., 2018).

Many computational methods of GP have been proposed and GBLUP is the most widely used (Meuwissen et al., 2001; Daetwyler et al., 2013; Mouresan et al., 2019). For conventional GBLUP, restricted maximum likelihood (REML) is often used to estimate heritability, and its computational complexity is cubic of the training population size (Xu et al., 2014). The fact that the inverse of the genomic relationship matrix (GRM) is essential when estimating the heritability in REML and calculating the best linear unbiased prediction (BLUP) contributes to the decrease of the computational efficiency of GBLUP when the size of the training population increases. To improve computational efficiency, methods such as IBS-based HE regression, algorithm for proven and young (APY), updating the inverse, recursive algorithm, spectral decomposition, and the preconditioned conjugate gradient (PCG) algorithm are employed (Kang et al., 2008; Legarra and Misztal, 2008; Misztal et al., 2009; Endelman, 2011; Faux et al., 2012; Meyer et al., 2013; Chen, 2014; Misztal, 2016; Liu and Chen, 2017; Masuda et al., 2017; Vandenplas et al., 2018; Vandenplas et al., 2020). In particular, PCG solves mixed model equations (MMEs) *via* iteration instead of by directly inversing the GRM. Recently, we proposed a fast GP method (SHEAPY) combining randomized Haseman–Elston regression (RHE-reg) and a modified APY (Liu and Chen, 2022). In the SHEAPY, RHE-reg is used to estimate heritability because of its high computational speed. In this study, we continue to combine it with PCG, calculating marker values to develop a new GP method (RHEPCG), which can significantly improve computational efficiency without the direct inverse of GRM.

## Materials and methods

### Genetic model and the linear system of MMEs

Herein, we only focus on additive effects, and the basic model is described as:


, (1) y=Xθ+Zu+e

in which  *y* is the *n*×1 vector of the standardized phenotypic values; **θ** is a fixed effect; **X** is the *n*×1 vector of the incidence; ***Z*** is the *n*×*m* matrix of the standardized genotypic values; **u** is the *m*×1 vector of SNP marker effects; and **e** is the *n* × 1 vector of the residual error.

On the basis of the above genetic model of additive effect, the linear system of MMEs was as follows:


, (2) $$\left[\begin{array}{cc} X^{T}X & X^{T}Z\\ Z^{T}X & Z^{T}Z+I\frac{\sigma_{e}^{2}}{\sigma_{g}^{2}} \end{array}\right]\left[\begin{array}{c} \hat{\theta }\\ \hat{u} \end{array}\right]=\left[\begin{array}{c} X^{T}y\\ Z^{T}y \end{array}\right]$$

in which I is the identity matrix, $\sigma_{g}^{2}$ is the variance of SNP marker effects, and $\sigma_{e}^{2}$ is the residual variance.

To solve MMEs, randomized HE-reg based on IBS was used to estimate heritability, which was then introduced into Equation 2 with residual variance. Then, PCG was used to solve Equation 2 to obtain marker values.

### Estimating heritability *via* randomized HE-reg based on IBS

IBS-based RHE regression is a method of moment that can reduce computational time and memory to $O\left(\frac{nmk}{\max\left(log_{3}^{\left(n\right)}log_{3}^{\left(m\right)}\right)}+nm\right)$ and *O*(*nm*) , respectively ( *n* , m , and k represent the number of samples, the number of markers, and the length of random vector, respectively) (Wu and Sankararaman, 2018; Liu and Chen, 2022). Here, it was used to estimate the heritability:


(3) $$y_{i}y_{i\;}=b_{0}+b_{1}\omega_{ij}+e$$

in which *y*_*i*_ and *y*_*j*_ represent the phenotypic values of individuals *i* and. from the training population; *b*_0_ is the intercept; *b*_1_ is the regression coefficient; is the genetic relatedness ( $\omega_{ij}=\frac{\text{ZiZj}"}{\text{m}}$ ) between a pair of individuals *i* and *j*; *z_i_* and *z_j_* are the genotype vector of individuals *i* and *j*; and is residual error. For a trait, its phenotypic variance is ${\hat{\sigma }}_{y}^{2}=\frac{\sum_{i=1}^{n}y_{i}^{2}-n{\bar{y}}^{2}}{n-1}$ , its additive genetic variance is ${\hat{\sigma }}_{g}^{2}={\hat{b}}_{1}$ , and its error variance is ${\hat{\sigma }}_{e}^{2}={\hat{\sigma }}_{y}^{2}-{\hat{\sigma }}_{g}^{2}$ . The computational equation of ${\hat{\sigma }}_{g}^{2}$ and ${\hat{\sigma }}_{e}^{2}$ is described as:


(4) $$\left[\begin{array}{cc} tr\left[\omega^{2}\right] & tr\left[\omega \right]\\ tr\left[\omega \right] & n \end{array}\right]\left[\begin{array}{c} {\hat{\sigma }}_{g}^{2}\\ {\hat{\sigma }}_{e}^{2} \end{array}\right]=\left[\begin{array}{c} y^{\text{T}}\omega y\\ y^{\text{T}}y \end{array}\right],$$

in which $\omega =\frac{ZZ^{\prime }}{m}$ corresponds to the genomic related matrix between individuals and $\hat{tr\left[\omega \right]}=n$ . To accelerate computational efficiency, $\hat{tr\left[\omega^{2}\right]}$ was calculated *via* a randomized estimation. The equation is as below:


(5) $$\hat{tr\left[\omega^{2}\right]}=\frac{1}{s}\frac{1}{m^{2}}\sum_{s=1}^{S}(w_{s}^{'}ZZ^{T})\left(ZZ^{T}w_{s}\right).$$

In the equation, *S* represents the rounds of randomization implemented, and was set as 5 throughout the study; each entry of ***w*** *_s_* comes from a standard normal distribution *N* (0,1).

### Preconditioned conjugate gradient (PCG) algorithm

When the MMEs are described as **Ax** = **b**, in which **A** is the coefficient matrix, **x** is the vector of solutions, and **b** is the right-hand side, the PCG is used to solve the linear system of MMEs and compute marker effects. As it does not need to invert GRM like conventional methods, a much higher efficiency can be achieved (Vandenplas et al., 2019). Its code is as follows (Tsuruta et al., 2001; Vandenplas et al., 2018):

When ***n* = 0**,

*x*_0_=1 ; *e*_0_=0 ;  *α*_0_=1 ( 1 is a vector of containing 1.);


; $$r_{0}=b-Ax_{0}$$

; $$p_{0}=M^{-1}r_{0}$$

When *n*=1, 2…..,


; $$w_{n}=M^{-1}p_{n-1}$$

; $$\alpha_{n}=p_{n-1}^{'}w_{n}$$

; βn=αnαn−1

; $$\alpha_{n-1}=\alpha_{n}$$

; $$e_{n}=w_{n}+e_{n-1}\beta_{n}$$

; $$q_{n}=Ae_{n}$$

; ϵn=pn−1'wnen'qn

; $$x_{n}=x_{n-1}+e_{n}\epsilon_{n}$$

$$r_{n}=r_{n-1}-\epsilon_{n}q_{n}$$

Until convergence.

End.

Here, $A=\left[\begin{array}{cc} X^{T}X & X^{T}Z\\ Z^{T}X & Z^{T}Z+I\frac{\sigma_{e}^{2}}{\sigma_{g}^{2}} \end{array}\right]$ , *M* is the preconditioner matrix, and **M =** diag(**A**); *r* , *p* , and *w* are vectors, $\text{x}=\left[\begin{array}{c} \hat{\theta }\\ \hat{u} \end{array}\right]$ , and $b=\left[\begin{array}{c} X^{T}y\\ Z^{T}y \end{array}\right]$ . To solve MMEs, ${\hat{\sigma }}_{g}^{2}$ *via* RHE regression based on IBS and ${\hat{\sigma }}_{e}^{2}$ are introduced into **A** matrix.

### Simulated data

The F2 population was simulated to evaluate the performance and cost time of GBLUP and RHEPCG. We simulated a chromosome with a length of 2,000 cM (the recombination rate was *c* between the *i^th^* and (*i*+1)^*th*^ markers), and all markers in this chromosome were defined as QTL, the effects of which followed a standard normal distribution. A series of different training population sizes (1,000, 1,200, 2,000, 6,000, 10,000, 15,000, and 20,000), candidate population sizes (100, 200, 300, and 400), and heritability (0.2, 0.4, 0.6, 0.65, and 0.8) were simulated. Each simulation scenario included 10 replications.

### Real data

Two sets of data (*Arabidopsis thaliana* and *Sorghum bicolor*) were used to evaluate the predictive accuracy of GBLUP and RHEPCG. (1) An *A. thaliana* F2 population (P15) with 434 individuals derived from a cross between Br-0 and C24 was obtained from the study by Salomé et al. It consisted of a total of 233 SNP markers and seven traits, including DTF1 (days until visible flower buds in the center of the rosette), DTF2 (days until inflorescence stem reached 1 cm in height), DTF3 (days until first open flower), RLN (rosette leaf number), CLN (cauline leaf number), TLN (total leaf number: sum of RLN and CLN), and LIR1 (leaf initiation rate [RLN/DTF1]) (Salomé et al., 2011). (2) A *S. bicolor* RIL population with 399 individuals derived from a cross between *S. bicolor* BTx623 and *S. bicolor* IS3620C was obtained from the study by Kong et al. It consisted of a total of 381 bins and five traits, including PH (plant height), BTF (base to flag length), FTR (flag to rachis length), ND (number of nodes), and FL (days to flowering). The phenotype data were obtained from the University of Georgia Plant Science Farm, Watkinsville, GA, USA on May 10, 2011 (Kong et al., 2018).

### Implementation and computations

The GBLUP and RHEPCG were written in R language (R Core Team, 2017) and run on a server of the CentOS Linux operating system (Intel (R) Xeon (R) CPU E7-4870 @2.40GHz) with 80 CPUs and 755G memory. The RHEPCG program is available from the authors. The squared correlation coefficient (*r^2^*) between the phenotypes and the predicted genotypic values were defined as the prediction accuracy.

## Results and discussion

### Comparison of GBLUP and RHEPCG in simulated F2 population studies

A series of simulations of the F2 population at different levels of parameters, including training population size, candidate population size, and heritability were used to assess the estimated heritability, predictive accuracy, and consumption time of GBLUP and RHEPCG.

**Table 1** shows the predictive accuracy and computational time of the GBLUP and RHEPCG at different training population sizes (1,000, 2,000, 6,000, 10,000, 15,000, and 20,000). As the training population size increased, both methods demonstrated an obvious uptrend of predictive accuracy. When the training population size was 1,000, RHEPCG was slightly better than GBLUP in predictive accuracy ( $r_{RHEPCG}^{2}=0.597\pm 0.007$ vs $r_{GBLUP}^{2}=0.541\pm 0.027$ ), but when the training population size was 10,000, an opposite result was achieved ( $r_{GBLUP}^{2}=0.640\pm 0.014$ vs $r_{RHEPCG}^{2}=0.607\pm 0.023$ ). In other conditions, both methods performed similarly (for example, when a training population size was 20,000 $r_{GBLUP}^{2}=0.653\pm 0.017$ vs. $r_{RHEPCG}^{2}=0.648\pm 0.021$ ). With the enlargement of the training population, the predictive accuracy approximated the true heritability, which as some studies have demonstrated, is the upper bound of predictive accuracy (de los Campos et al., 2013; Liu and Chen, 2018). Meanwhile, RHEPCG was significantly faster than GBLUP (for example, when a training population size was 20,000 *T*_*GBLUP*_=53666s vs *T*_*RHEPCG*_=1237s ). When the training population size was 1,000, 2,000, 6,000, 10,000, 15,000, and 20,000, the computational time of GBLUP was 4, 8, 9, 15, 28, and 43 times that of RHEPCG, respectively. In other words, the larger the training population size, the more obvious the advantage of the computational efficiency of RHEPCG becomes.

**Table 1:** Comparison of the estimated heritability, predictive accuracy, and computational time of GBLUP and RHEPCG at the different training population sizes based on 10 simulations in the *Arabidopsis thaliana* F2 population.

Training size	GBLUP			RHEPCG		
	${\hat{h}}_{GBLUP}^{2}$	$r_{GBLUP}^{2}$	Average time of each simulation (s)	${\hat{h}}_{RHEPCG}^{2}$	$r_{RHEPCG}^{2}$	Average time of each simulation (s)
1,000	0.718±0.055	0.541±0.027	135	0.637±0.042	0.597±0.007	31
2,000	0.648±0.029	0.576±0.011	465	0.625±0.051	0.570±0.010	58
6,000	0.668±0.052	0.615±0.011	1,580	0.621±0.055	0.594±0.013	179
10,000	0.680±0.042	0.640±0.014	7,932	0.600±0.043	0.607±0.023	526
15,000	0.650±0.038	0.651±0.013	22,576	0.724±0.059	0.643±0.024	820
20,000	0.698±0.042	0.653±0.017	53,666	0.728±0.043	0.648±0.021	1,237

The training population sizes were 1,000, 2,000, 6,000, 10,000, 15,000, and 20,000; the candidate population size was 100; the length of the chromosome was 2,000; heritability (h^2^) was set as 0.65; the recombination rate ^©^ was set as 0.01. ${\hat{h}}_{GBLUP}^{2}$ and ${\hat{h}}_{RHEPCG}^{2}$ represent the estimated heritability via GBLUP and RHEPCG, respectively; $r_{GBLUP}^{2}$ and $r_{RHEPCG}^{2}$ represent the squared correlation coefficient between the phenotypes and the predicted genotypic values and are defined as the prediction accuracy; the values after ± represent the corresponding standard error.

In **Table 2**, the predictive accuracy of both methods was similar at different candidate population sizes (100, 200, 300, and 400), which means the latter has no significant impact on the former. **Table 3** shows that the predictive accuracy of GBLUP and RHEPCG increased when heritability varied from 0.2 to 0.4, 0.6, and 0.8. According to further analysis, the correlation between the estimated heritability and the predictive accuracy was 0.999 for both GBLUP and RHEPCG (*P*_*Two*−*tailed*_=0.001 ), and our results are consistent with Daetwyler et al. (2008) in that heritability can significantly influence predictive accuracy.

**Table 2:** Comparison of the predictive accuracy of GBLUP and RHEPCG at the different candidate population sizes based on 10 simulations in the *Arabidopsis thaliana* F2 population.

Candidate population	$r_{GBLUP}^{2}$	$r_{RHEPCG}^{2}$
100	0.581 ± 0.022	0.560 ± 0.016
200	0.561 ± 0.015	0.568 ± 0.016
300	0.561 ± 0.014	0.578 ± 0.008
400	0.580 ± 0.008	0.571 ± 0.011

The training population sizes was 1,200; the candidate population sizes were 100, 200, 300, and 400; the length of the chromosome was 2,000; heritability (h^2^) was set as 0.65; the recombination rate (c) was set as 0.01. $r_{GBLUP}^{2}$ and $r_{RHEPCG}^{2}$ represent the squared correlation coefficient between the phenotypes and the predicted genotypic values and are defined as the prediction accuracy; the values after ± represent the corresponding standard error.

**Table 3:** Comparison of the estimated heritability and predictive accuracy of GBLUP and RHEPCG at different levels of heritability based on 10 simulations in the *Arabidopsis thaliana* F2 population.

*h* ^2^	GBLUP		RHEPCG	
	${\hat{h}}_{GBLUP}^{2}$	$r_{GBLUP}^{2}$	${\hat{h}}_{RHEPCG}^{2}$	${\hat{h}}_{GBLUP}^{2}$
0.2	0.220 ± 0.014	0.165 ± 0.021	0.203 ± 0.009	0.127 ± 0.017
0.4	0.460 ± 0.021	0.346 ± 0.027	0.406 ± 0.014	0.304 ± 0.018
0.6	0.708 ± 0.032	0.535 ± 0.025	0.611 ± 0.019	0.506 ± 0.016
0.8	0.910 ± 0.034	0.667 ± 0.032	0.817 ± 0.024	0.726 ± 0.012

The training population sizes was 1,200; the candidate population size was 100; the length of the chromosome was 2,000; heritability (h^2^) was set as 0.2, 0.4, 0.6, and 0.8; the recombination rate (c) was set as 0.01. ${\hat{h}}_{GBLUP}^{2}$ and ${\hat{h}}_{RHEPCG}^{2}$ represent the estimated heritability via GBLUP and RHEPCG, respectively; $r_{GBLUP}^{2}$ and $r_{RHEPCG}^{2}$ represent the squared correlation coefficient between the phenotypes and the predicted genotypic values and are defined as the prediction accuracy; the values after ± represent the corresponding standard error.

Currently, IBS-based RHE regression is used to estimate gene-environmental heritability and multi-trait genetic correlation (Kerin and Marchini, 2020; Wu et al., 2022). Therefore, RHEPCG can also be applied to such data *via* the incorporation of these effects into the model in the future.

### Comparison of GBLUP and RHEPCG in studies of the *A. thaliana* F2 and *S. bicolor* RIL populations

A comparison of GBLUP and RHEPCG based on seven traits of the *A. thaliana* F2 population was performed in this study. **Table 4** shows a significant difference between the estimated heritability *via* GBLUP and that *via* RHEPCG in seven traits of *A. thaliana* F2 (P15). Meanwhile, the seven traits were used to evaluate the predictive accuracy of GBLUP and RHEPCG (**Table 4**). The two methods showed similar predictive accuracy in six traits: DTF1, DTF2, DTF3, CLN, TLN and LIR1 (for example, the predictive accuracies of DTF1 were $r_{GBLUP}^{2}=0.383\pm 0.017$ and $r_{RHEPCG}^{2}=0.368\pm 0.024$ ). RHEPCG was significantly better than GBLUP for RLN (the predictive accuracies of RLN were $r_{GBLUP}^{2}=0.555\pm 0.046$ and $r_{RHEPCG}^{2}=0.640\pm 0.015$ ).

**Table 4:** Comparison of the predictive accuracy between GBLUP and RHEPCG in seven traits from the *Arabidopsis thaliana* F2 (P15) population based on 10 simulations.

Trait	Training	Candidate	GBLUP		RHEPCG	
			${\hat{h}}_{GBLUP}^{2}$	$r_{GBLUP}^{2}$	${\hat{h}}_{RHEPCG}^{2}$	$r_{RHEPCG}^{2}$
DTF1	300	133	0.731 ± 0.025	0.383 ± 0.017	0.323 ± 0.034	0.368 ± 0.024
DTF2	300	134	0.604 ± 0.035	0.406 ± 0.024	0.389 ± 0.029	0.401 ± 0.023
DTF3	300	134	0.645 ± 0.033	0.351 ± 0.024	0.311 ± 0.030	0.321 ± 0.025
RLN	300	131	0.923 ± 0.025	0.555 ± 0.046	0.524 ± 0.030	0.640 ± 0.015
CLN	300	130	0.570 ± 0.017	0.350 ± 0.017	0.282 ± 0.019	0.342 ± 0.018
TLN	300	130	0.910 ± 0.022	0.572 ± 0.029	0.421 ± 0.028	0.619 ± 0.011
LIR1	300	131	0.449 ± 0.033	0.208 ± 0.012	0.162 ± 0.011	0.197 ± 0.017

${\hat{h}}_{GBLUP}^{2}$ and ${\hat{h}}_{RHEPCG}^{2}$ represent the estimated heritability via GBLUP and RHEPCG, respectively; $r_{GBLUP}^{2}$ and $r_{RHEPCG}^{2}$ represent the squared correlation coefficient between the phenotypes and the predicted genotypic values and are defined as the prediction accuracy; the values after ± represent the corresponding standard error.

In addition, the predictive accuracy of GBLUP and RHEPCG was evaluated based on five traits of the *S. bicolor* RIL population (**Table 5**). The estimated heritability of PH, BTF, FTR, and ND *via* GBLUP differed significantly from that *via* RHEPCG, and the two methods had similar predictive accuracy for PH, BTF, FTR, and FL (for example, the predictive accuracies of PH were $r_{GBLUP}^{2}=0.289\pm 0.011$ and $r_{RHEPCG}^{2}=0.257\pm 0.016$ ), and the predictive accuracy of GBLUP was significantly superior to that of RHEPCG for ND (the predictive accuracies of ND were $r_{GBLUP}^{2}=0.306\pm 0.015$ and $r_{RHEPCG}^{2}=0.197\pm 0.039$ ).

**Table 5:** Comparison of the predictive accuracy between GBLUP and RHEPCG in five traits from the *Sorghum bicolor* RIL population based on 10 simulations.

Trait	Training	Candidate	GBLUP		RHEPCG	
			${\hat{h}}_{GBLUP}^{2}$	${\hat{r}}_{GBLUP}^{2}$	${\hat{h}}_{RHEPCG}^{2}$	${\hat{r}}_{RHEPCG}^{2}$
PH	300	88	0.653 ± 0.013	0.289 ± 0.011	0.331 ± 0.038	0.257 ± 0.016
BTF	300	88	0.619 ± 0.011	0.335 ± 0.019	0.436 ± 0.047	0.302 ± 0.017
FTR	300	88	0.337 ± 0.008	0.182 ± 0.018	0.493 ± 0.056	0.155 ± 0.026
ND	300	88	0.490 ± 0.011	0.306 ± 0.015	0.747 ± 0.065	0.197 ± 0.039
FL	300	93	0.629 ± 0.014	0.371 ± 0.014	0.622 ± 0.043	0.393 ± 0.031

${\hat{h}}_{GBLUP}^{2}$ and ${\hat{h}}_{RHEPCG}^{2}$ represent the estimated heritability via GBLUP and RHEPCG, respectively; ${\hat{r}}_{GBLUP}^{2}$ and ${\hat{r}}_{RHEPCG}^{2}$ represent the squared correlation coefficient between the phenotypes and the predicted genotypic values and are defined as the prediction accuracy; the values after ± represent the corresponding standard error.

These results show that GBLUP and RHEPCG have different estimated heritability in some traits of *A. thaliana* and *S. bicolor*. According to Chen (2016), strong selection can lead to differences in the estimated heritability *via* LMM and HE, and therefore, these traits are very likely to have undergone strong selection. In the future, we will investigate the influence of strong selection on predictive accuracy.

## Conclusion

We present a new computing method of genomic prediction (RHEPCG) that does not require direct inversion of the GRM. Compared with GBLUP, it can significantly reduce computational time while maintaining predictive accuracy.

## Data availability statement

The original contributions presented in the study are included in the article/supplementary material. Further inquiries can be directed to the corresponding author.

## Author contributions

HLL conceived and performed the study, interpreted the results, and wrote the manuscript. HL and CX interpreted the results and wrote the manuscript. All authors contributed to the article and approved the submitted version.
